# Theranostic Near-Infrared Monoamine Oxidase Inhibitor (NMI) Protein Binding Interactions with MAOA and Albumin

**DOI:** 10.1007/s11095-025-03827-1

**Published:** 2025-02-04

**Authors:** Ronald W. Irwin, Unnati H. Shah, Shivani Soni, Heinz Josef Lenz, Jean C. Shih

**Affiliations:** 1 Department of Pharmacology and Pharmaceutical Sciences, USC Alfred E. Mann School of Pharmacy and Pharmaceutical Sciences, University of Southern California, Los Angeles, CA USA; 2 Norris Comprehensive Cancer Center, Keck School of Medicine of the University of Southern California, Los Angeles, CA USA

**Keywords:** albumin, colorectal cancer, glioblastoma, MAOA, monoamine oxidase, theranostic

## Abstract

**Purpose:**

The protein binding interactions of near-infrared monoamine oxidase inhibitor (NMI) are reported here.

**Methods:**

NMI-bound proteins were examined by fluorescent SDS-PAGE and mass spectrometry using tumor tissues from brain and colon cancer mouse models.

**Results:**

This study shows protein interactions with NMI, a chemical conjugate of MAOA inhibitor clorgyline and tumor-seeking dye, MHI-148. NMI fluorescence in MAOA knock-out (KO) mice was significantly lower compared to WT mice, including whole animal, organs, and tissue lysates which indicated that NMI binds to MAOA. Pure recombinant MAOA protein was detectable as a single fluorescent band that migrated at ~ 65kD. NMI inhibited MAOA activity (IC_50_ 1–5 µM). In a glioma mouse model, NMI targeted specifically to tumor with high contrast to adjacent normal brain, shown by a 65 kD protein band. Recent studies demonstrated heptamethine cyanine dyes (e.g., MHI-148) interact with serum albumin, contributing to tumor uptake and cancer cell internalization. Our study shows NMI binds to albumin but highly prefers MAOA, providing a plausible mechanism for systemic drug delivery via serum albumin to the tumor target and subsequent MAOA inhibition. Further studies in a colon cancer mouse model found the ~ 65 kD SDS-PAGE band, bound to NMI, contained both MAOA and albumin proteins by mass spectrometry.

**Conclusion:**

NMI was shown to interact with MAOA and the blood carrier protein, albumin. This study provides insights for drug delivery and protein target specificity of NMI to image and treat cancer.

## Introduction

Monoamine oxidases (MAOs), located on the mitochondrial outer membrane, produce H_2_O_2_ via oxidative deamination of monoamine neurotransmitters and have been implicated in various diseases, including cancer [1]. Research advances have implicated abnormal MAOA activity in aggressive cancers, including prostate [2–5], lung [6], brain [7], colon [8], melanoma [9], Hodgkin lymphoma [10], and breast cancer [11]. Near Infrared Monoamine Oxidase Inhibitor (NMI) is a chemical conjugate of the selective MAOA inhibitor clorgyline and MHI-148, a near-infrared (NIR) fluorescent heptamethine cyanine dye (HMCD) molecule. NMI functions to selectively target tumors for localized therapy, non-invasive biomedical imaging, and to identify tumor margins during surgical excision [7, 12, 13]. NMI has been investigated as a potential stand-alone or combination therapy for treatment-resistant prostate cancer [14], glioma [7, 15], and other cancers with an efficacious range of 1–10 µM to inhibit cancer cell growth *in vitro* and 5 mg/kg to inhibit tumor growth in mice. Anticancer efficacy of NMI *in vitro* and *in vivo*, surpasses its parent compound, clorgyline, thus NMI efficacy may uniquely include other mechanisms of actions beyond MAO inhibition [7, 14]. Understanding specific protein binding interactions of NMI with MAOA and any yet identified proteins *in vivo* could prove important for safety and efficacy of NMI as it advances towards clinical studies.

Recent evidence suggests that HMCDs bind to serum albumin before cancer cell internalization [16]. Albumin has been reported to naturally accumulate in various types of solid tumors, including sarcomas, lung cancers, and glioblastoma multiforme (GBM). In these tumors, albumin, a nutrient carrier itself, serves as a primary nutrient source and can be endocytosed by cancerous cells [17]. Several groups have successfully utilized albumin-based drug delivery systems, such as Abraxane®, which is the first albumin-based drug approved in oncology [18]. The albumin-HMCD adducts can passively accumulate in tumors via the enhanced permeability and retention (EPR) effect. Additionally, the albumin-HMCD adducts can be actively taken up by cancer cells through various receptors that import albumin (gp60, gp18, and gp30 cell surface glycoproteins, megalin/cubilin complex, secreted protein acidic and rich in cysteine (SPARC), and FcRn), often overexpressed in cancer cells [19]. In cell culture experiments, fluorescently labeled albumin can permeate into cancer cells and colocalize with the HMCD NIR fluorescence. This suggests that both covalent and non-covalent complexes formed with albumin are associated with cellular import, and both forms persist within cancer cells [20]. According to studies, for HMCDs or their conjugates to become active and exert their biological effects, they must be separated from the albumin to which they are bound. Research suggests that the acidic environment found in tumors may help with this separation process by causing a cleavage of the covalent adducts from albumin [21]. Overall, albumin plays a crucial role in transporting HMCDs, facilitating their accumulation for tumor imaging and targeted drug delivery.

In this study, we investigated the binding of the theranostic molecule NMI to MAOA and albumin and discuss the implications of these molecular interactions. Such insights significantly contribute to our understanding of the underlying mechanism and hold promise for translation into clinical trials.

## Methods

### NMI Dose Preparation

NMI chemical synthesis has been described previously [14]. For *in vitro* studies, NMI was prepared in dimethyl sulfoxide (DMSO) at 1 × 10^–2^ M and then further serial-diluted in phosphate buffered saline, pH 7.4 (PBS, Gibco) or polyethylene glycol-400 (PEG; Sigma) PEG 66% in saline. For *in vivo* studies, NMI was prepared at a concentration of 3 mg/mL in a formulation of PEG-400 (Sigma), 66% volume to volume, in sterile saline for injection and vortexed on ice to solubilize.

### Animal Models

#### MAOA Knock-Out (KO) and Wildtype MAOA Mice

MAOA KO (KO) mice were first created and bred in our laboratory [22]. KO mice with a point mutation similar to human MAOA deficiency (Brunner syndrome) and their wild-type (WT) littermates were generated and genotyped as described elsewhere [23]. This strain carries a spontaneous mutation *Maoa*^*K284stop*^, a transversion that arises at nucleotide 863 in exon 8 creating a stop codon at amino acid residue 284 rather than a lysine, in the X-linked *Maoa* gene which abolishes gene function in all tissues [24, 25]. NMI formulation was administered intravenously (IV) into the lateral tail vein via bolus injection, 5 mg/kg (42ul/25 g), and mice were imaged 1 h and 24 h after a single dose. Brain and liver were removed at 24 h, imaged, and tissue samples flash-frozen for NMI-binding protein studies. Adult mice were between 2 and 3 months of age for these studies and housed in the animal research facility at University of Southern California (USC). All animal experiments were conducted according to the USC’s Animal Ethics Committee approvals. Mice were fed a normal diet of standard chow and housed under standard controlled conditions of temperature, humidity, and light; water and food were available *ad libitum*.

#### Intracranial Glioma Mouse Model

A syngeneic immunocompetent glioma mouse model was used [26]. Intracranial implantation of GL26 glioma cells was conducted in male C57BL/6 J WT mice. Mice were anesthetized and surgically implanted following approved standard procedures as described previously [15]. Briefly, the head was shaved, sagittal skin incision made with scalpel, and the skull exposed. The animal was positioned into a stereotactic frame. A Dremel drill was used to make a hole at approximately 3 mm lateral and 1 mm posterior from the intersection of the coronal and sagittal sutures (bregma). Luciferase-positive GL26 glioma cells (5 × 10^3^) were injected using a Hamilton syringe at a depth of 3 mm in a volume of 5 μl. Tumor developed within 1 week and was confirmed for appropriate size and location by luciferase expression prior to NMI treatment. A single dose of NMI 5 mg/kg IP was administered and 1 week later, brain and tumor were removed for NMI-binding protein studies.

#### Subcutaneous Syngeneic Colon Cancer Mouse Model

For syngeneic colorectal cancer (CRC) mice model, mismatch repair deficient (dMMR)/microsatellite instability-high (MSI-H) MC-38 colon cancer cells were used. 5 × 10^6^ MC38 cells in 100 µl PBS were injected subcutaneously into the right flank of 7–8 weeks old C57BL/6 J WT female mice. Once the tumor was visible around 7–10 d post-injection, mice were randomized and received the following treatments: vehicle (67% PEG400 and 33% saline), NMI (5 mg/kg; every other day; IP) for 21 d. Tumors were collected 24 h after last treatment and lysed for protein analysis.

### Optical Imaging

Mice were imaged for NIR signal (emission: 780 nm, excitation: 845 nm) with a photographic image overlay using the IVIS Lumina Series III optical imaging system (Perkin Elmer); images were analyzed using Living Image software (Perkin Elmer). Imaging data presented as total flux which equals the radiance (photons/sec) in each pixel summed or integrated over the ROI area (cm2) x 4π. During imaging, mice were maintained in an anesthetized state with isoflurane, 2.5%.

### Pure Protein Samples

Pure protein bovine serum albumin (BSA; Sigma, A9418) was used for *in vitro* NMI binding studies. Human serum albumin shares 76% sequence homology with BSA and 72% sequence homology with mouse serum albumin [27, 28]. Importantly for HMCD binding, the free thiol associated with Cys34 of mouse, bovine, and human albumin is conserved along with 17 conserved intradomain disulfide bridges that form hydrophobic pockets [27, 29, 30]. Thus, BSA is commonly used as a substitute for human serum albumin.

Recombinant human MAOA (M7316-1VL) was used for *in vitro* NMI binding studies and enzyme activity assay. Human and mouse MAOA have over 87% amino acid identity and similar 3-D structure, including the hydrophobic cavity in front of the flavin cofactor active site [1, 31, 32]. Thus, we used purified human MAOA to support NMI binding studies in the mouse model. Both the purified human enzyme and mouse tissue lysates showed a distinct ~ 65 kD fluorescent band by SDS-PAGE. Pure protein samples were prepared in phosphate buffered saline (PBS), pH 7.4 at various concentrations.

### MAOA Catalytic Activity Assay

Dose-dependent inhibition of MAOA was determined with 0.25 µg MAOA pure protein using MAO-Glo™ assay (Promega Corporation, Wisconsin) as per the manufacturer's instructions and literature search [33]. Equal amounts of the reaction sample (22.5 µl MAOA enzyme in buffer and 2.5 µl test inhibitor, total 25 µl) and the MAO substrate (1:25 dilution of the provided stock MAO substrate to determine MAOA activity, total 25 µl) were incubated for one hour at 37 ºC. 50 µl of the luciferin detection reagent was then added after the incubation and the reaction mixture was allowed to stand for 10 min. The luminescent signal was measured and recorded, and the activity was expressed as NET RLU/ µg protein/ hr. Log-dose inhibition curves were plotted and the 50% inhibitory concentration (IC_50_) values (μM) were expressed as the mean ± SD (*n* = 3–5).

### SDS-PAGE Analysis of NMI Interacting Proteins

Protein extracts were separated by SDS-PAGE, 10% polyacrylamide gel. Protein concentration was determined by BCA reagent standard assay. Samples containing 20–30 µg protein from mouse tissue lysates or 1–5 µg pure protein in PBS were prepared on ice with standard 4X blue loading buffer and electrophoresed in Tris/glycine running buffer with constant 100 V for 1.5 h. Gels were NIR-imaged with iBright, excitation (745–765 nm) and emission (810–850 nm) with exposure time 10–30 s. Molecular standard marker was used to estimate relative mass. Gel results representative of 2 or more replicate experiments.

### Mass Spectrometry Protein Analysis

Tumor tissue lysates from MC-38 colon cancer mice treated with NMI or vehicle were loaded (20 µg) and separated by SDS-PAGE. The NMI binding protein band ~ 65 kD was excised from the gel and sent for mass spectrometry analysis at Poochon Scientific (Frederick, MD). Gel samples were digested using a standard trypsin/lysC method, the digested peptides were then concentrated, desalted, and reconstituted for LC–MS/MS. Analysis was conducted by Orbitrap Exploris 240 Mass Spectrometer and Dionex UltiMate 3000 RSLCnano system (Thermo Scientific) operated in the data-dependent mode which automatically switches between full scan MS and tandem MS/MS acquisition modes. Mass spectrometry raw data files were analyzed by Proteome Discoverer 2.5, based on the SEQUEST algorithm, and selecting the mouse protein sequences database downloaded from NCBI and UniProtKB.

### Statistics

Data are presented as the mean ± SD, where indicated. All comparisons were done in at least triplicate and were analyzed by unpaired two-tailed Student’s *t*-test or to compare multiple groups means, one way analysis of variance ANOVA and Tukey’s post hoc test. A *p*-value ≤ 0.05 was considered statistically significant.

## Results

### NMI Signal Highly Expressed in WT but not MAOA KO Mice

**Fig. 1:**
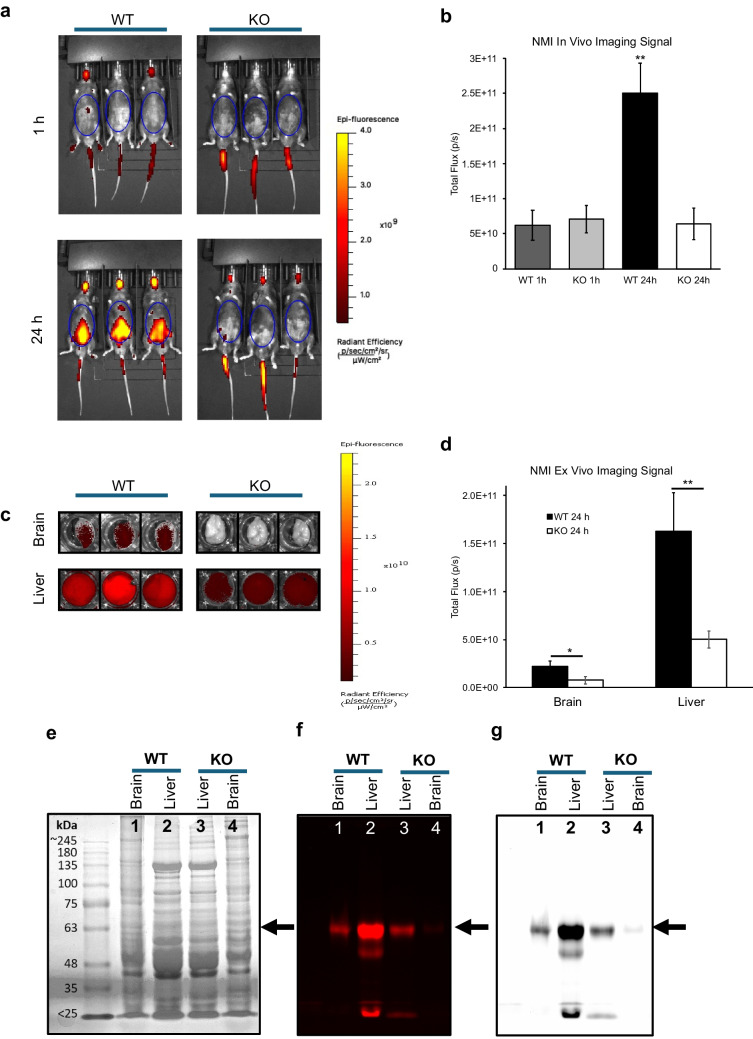
NMI was administered as single bolus dose (5 mg/kg) by intravenous (i.v.) route to C576BL/6 J (WT) and MAOA knock-out (KO) mice and imaged with IVIS system in near-infrared range to determine differences in uptake and biodistribution. (**a**) Whole animal *in vivo* comparison of NMI biodistribution in WT and KO mice at 1 h and 24 h. (**b**) Quantitation of total flux (photons/s) of total ventral (belly) fluorescent region of interest (ROI) at 1 h and 24 h (*n* = 3 per group). (**c**) Brain and liver organ comparison at 24 h. (**d**) Quantitation of total flux (photons/s) of organs *ex vivo* after 24 h (*n* = 3 per group). Brain and liver tissues were separated on SDS-PAGE gel and imaged with iBright. (**e**) Coomassie total protein stain and molecular size marker. (**f**) Near-infrared iBright image of gel showed major band at ~ 65 kD. (**g**) Black and white image of same gel.

As shown in Fig. 1a, near-infrared fluorescence uptake of NMI in live mice shows higher NMI fluorescence in WT compared to KO, 24 h after NMI injection. Figure 1b, the ventral region of interest (ROI), a general measurement of internal organ uptake was measured and showed a significantly fourfold increased peak signal observed in WT mice compared to KO at 24 h (WT: 2.51E + 11 ± 4.24E + 10 *vs* KO: 6.41E + 10 ± 2.24E + 10 p/s), (*p* < 0.005; *n* = 3 per group). One way ANOVA test was also significant, *p* = 0.0001 with Tukey’s post hoc analysis, *p* = 0.0002 for each group compared with WT 24 h. Ventral images were not significantly different between WT and KO at 1 h, both showing a low background reading (5E + 10 p/s) in the ROI similar to the value at 24 h for KO. We showed previously that within 1 h post-dose, NMI fluorescence reached an initial absorption phase peak that redistributes with increased ROI peak intensity by 24 h in WT [15]. *Ex vivo* organs were harvested at 24 h and quantified to understand biodistribution and retention of NMI, brain and liver are shown in Fig. 1c. Organ signal intensities reflected the whole animal imaging at 24 h, WT organs were significantly more intense than KO for brain (2.23E + 10 ± 5.66E + 09 *vs* 7.72E + 09 ± 3.62E + 09 p/s, *p* < 0.05) and liver (1.63E + 11 ± 4.02E + 10 *vs* 5.03E + 10 ± 8.83E + 09 p/s, *p* < 0.01), respectively (*n* = 3). Brain and liver tissue lysates were separated by SDS-PAGE. A distinct fluorescent band was evident in WT tissues migrating at ~ 65 kD but to a much lesser extent in KO tissues suggesting an NMI-MAOA protein interaction not present in KO, Fig. 1e-g. Brain KO tissue showed relatively no band as compared to the WT tissue, thus this band represents MAOA. Moreover, KO liver tissue had weaker intensity band compared to WT, again suggesting this band represents MAOA binding but to some extent another abundant liver or blood protein of similar molecular size as MAOA. In WT, NMI bands were much more robust in liver than brain. This MAOA tissue expression pattern is well-established, MAOA activity is relatively much higher in liver than brain [34–36]. Thus, these results suggest the ~ 65 kD fluorescent protein band bound to NMI largely represents MAOA in the WT tissues and not in KO tissues.

### NMI Binds to 65 kD Protein in Brain Tumor but not Surrounding Tissue

**Fig. 2:**
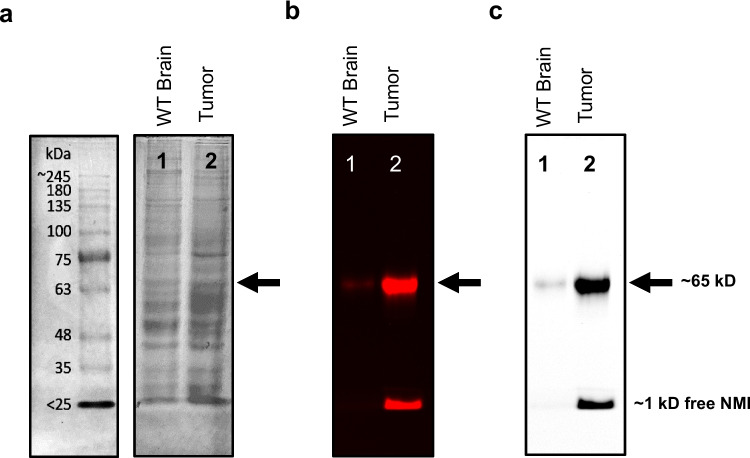
C576BL/6 J (WT) mice bearing intracranial glioma GL26 tumors were treated with NMI (5 mg/kg), normal adjacent brain and tumor samples were collected 1 week after a single dose. Protein lysates were separated by SDS-PAGE and imaged with iBright at NIR wavelength for analysis. (**a**) Coomassie total protein stain and molecular size marker. (**b**) Near-infrared iBright image of gel showed a major band at ~ 65 kD. Normal brain tissue surrounding the tumor (Lane 1) showed relatively no visible NMI band. Intracranially implanted GL26 glioblastoma tumor tissue (Lane 2) displayed a high-intensity NMI band. The fastest migrating band at gel front was attributed to ~ 1 kD representing the free NMI-amide molecule (formula weight 980.5) that separated from the protein sample during SDS-PAGE conditions. (**c**) Black and white image of same gel.

As shown in Fig. 2, SDS-PAGE gel revealed a strong protein binding interaction with NMI at ~ 65 kD in orthotopic GL26 glioblastoma tumor tissue lysate. Adjacent brain tissue from the same tumor-burdened mouse expressed much lower intensity compared to tumor sample. Additionally, in the tumor sample, more unbound or less tightly bound NMI was observed at the dye front under SDS-PAGE conditions. The robust fluorescence within the protein band of brain tumor tissue lysate indicates that systemic injection of NMI reaches the brain with protein binding specificity and accumulates in highly vascularized tumors rather than adjacent normal brain tissue, important parameters for both drug targeting and diagnostic imaging utility.

### NMI Binds to MAOA

**Fig. 3:**
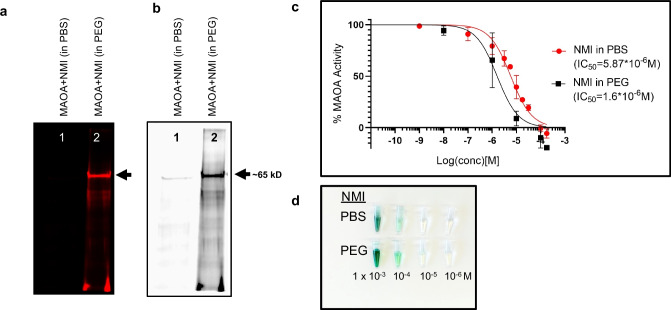
Purified recombinant human MAOA (7 µg) was incubated with NMI (10 µM in PBS *vs*. PEG) at 37 ºC for 1 h. After 1 h, 5 µg protein was loaded on SDS-PAGE. (**a**) Near-infrared iBright image of gel showed major band at ~ 60–65 kD which demonstrated MAOA and NMI binding. (**b**) Black and white image of same gel. (**c**) Purified recombinant human MAOA glo activity assay inhibition by NMI in PEG formulation improved solubility and improved potency (IC_50_ = 1.6 µM; black squares) of NMI in MAO glo activity assay compared to PBS formulation (IC_50_ 5.87 µM; red circles). As a control, the irreversible selective MAOA inhibitor clorgyline (IC_50_ 2.8 nM) was measured under the same conditions (not shown). The inhibitor potency of NMI was enhanced almost fourfold by improving NMI solubility in PEG-400 in aqueous reaction, mean IC_50_ of 3–5 separate experiments, (*p* < 0.05). (**d**) Visual representation of NMI serial-diluted range of 1 × 10^–3^ M to 1 × 10^–6^ M NMI in PBS (top) compared to NMI in PEG-400 66% (bottom) working solutions that were then tenfold diluted in assay buffer for MAO glo assay.

To investigate NMI’s interactions with MAOA, we performed a controlled protein binding experiment using pure human recombinant MAOA enzyme. In Fig. 3a, b, a single NMI-bound protein band was observed at ~ 65kD, within the predicted molecular size range of monomeric MAOA protein. This confirms that NMI binds to MAOA and the binding interaction was detectable under SDS-PAGE conditions at the NMI concentration (10 µM) expected to inhibit the active enzyme.

### Solubilizers Enhance NMI-MAOA Protein Interaction

Due to the hydrophobic structure of NMI, at millimolar concentrations it is only slightly soluble in aqueous PBS. PEG is often used as a non-ionic hydrophilic polymeric carrier for hydrophobic molecules to enhance aqueous solubility and dissolution characteristics. In this experiment, standard PBS serial-diluted NMI was compared to PEG-formulated NMI which resulted in greatly enhanced NMI interaction with MAOA protein (~ 2.5-fold increased band intensity), as shown in Fig. 3a, b. NMI dose–response inhibition of MAOA enzyme activity assay confirmed the PEG-enhanced binding interaction (Fig. 3c). Previously, we showed MAOA activity is inhibited by NMI and inhibited cell growth in various cancer cell cultures [7, 14, 37, 38]. NMI inhibited MAOA activity with an IC_50_ of 3.8 µM in LNCaP-derived C4-2B prostate cancer cells [14] and 5 µM in GL26 glioma cells [7]. Recombinant human MAOA enzyme (0.25 µg) was incubated with NMI at a series of concentrations. As expected, pure recombinant MAOA catalytic activity was inhibited by NMI with an IC_50_ of 5.87 µM. NMI solubilized in PEG significantly lowered the IC_50_ to 1.6 µM, a nearly fourfold increase in potency (*p* < 0.05). Using PEG to solubilize NMI demonstrated that the initial solubility of the inhibitor is an important experimental factor to optimize MAOA inhibition potency. In the gel Fig. 3a, b, MAOA was incubated with NMI 10 µM for 1 h and the 65 kD band was apparent and more pronounced in PEG-solubilized NMI. SDS-PAGE showed MAOA ~ 65 kD band when the NMI concentration was relatively high (10 µM), binding conditions similar to the MAOA inhibition assay (Fig. 3c) where during the 1 h activity assay, NMI 10 µM inhibited MAOA 60% (PBS) *vs* 91% (PEG).

### NMI Binds to Albumin

**Fig. 4:**
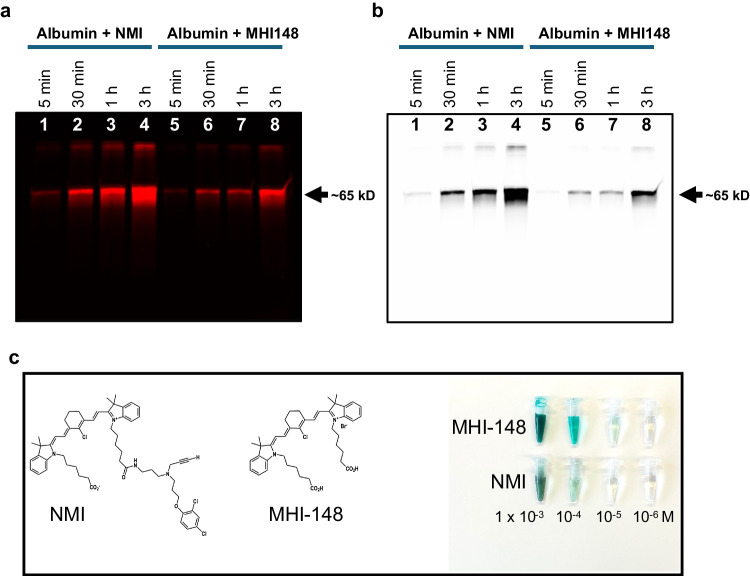
Albumin (3 µg in 24 µl) and NMI (1 µM) or MHI-148 (1 µM) were incubated for 5 min, 30 min, 1 h and 3 h at 37 ºC. After the incubation, 2 µg albumin protein was loaded on SDS-PAGE. Gel was imaged using iBright at NIR wavelength. (**a**) Near-infrared iBright image of gel showed major band at ~ 65 kD (monomer) and ~ 135 kD (dimer) with increased intensity at 3 h. (**b**) Black and white image of same gel. (**c**) The chemical structure of NMI (FW 980.55) and its NIR dye moiety MHI-148 (FW 764.23) and color photograph of their serial dilutions from a stock solution of 1 × 10^–2^ M in DMSO to a range of working solutions, 2.5 × 10^–3^ M to 1 × 10^–6^ M in PBS.

To determine the NMI binding interaction with albumin, the most abundant blood carrier, pure albumin was incubated with NMI at 5 min, 30 min, 1 h, and 3 h. In Fig. 4a, b, albumin (2 µg) and NMI of the same molar concentrations (1 µM each) were observed as a bright ~ 65 kD band, binding increased with incubation time. NMI-bound albumin aggregates (~ 135 kD) were also observed in the gel. It has been previously shown that the chlorine group of the MHI-148 dye (heptamethine cyanine dye chemical moiety of NMI) binds covalently to albumin at Cys34 [21]. NMI structure is comprised of MHI-148 dye and MAOA irreversible inhibitor, clorgyline, Fig. 4c. Thus, as reported for MHI-148, it was predicted that the MHI-148 HMCD moiety of NMI would bind to albumin to form a covalent adduct. NMI and MHI-148 both showed a ~ 65 kD band indicating that the HMCD moiety of NMI is involved in albumin binding.

### NMI Binding Affinity for MAOA and Albumin

**Fig. 5:**
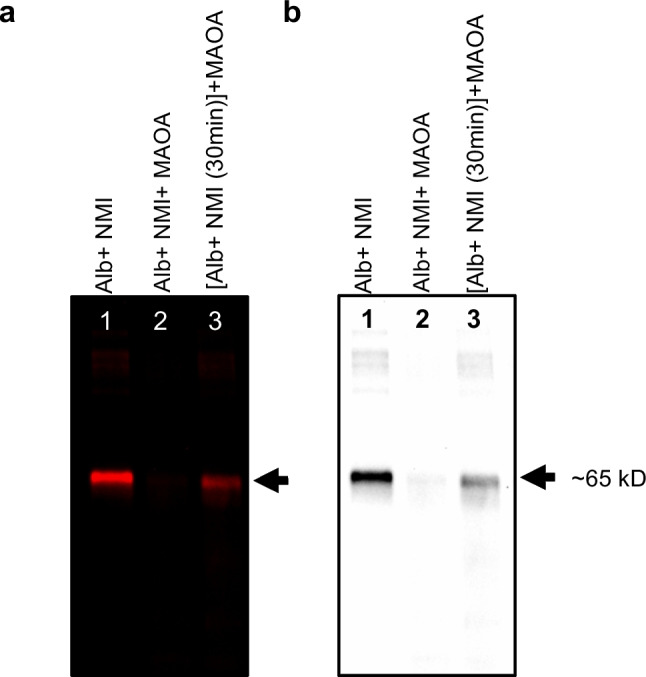
NMI (1 µM), MAOA (1 µM) and albumin (1 µM) were incubated in different combinations to understand the binding affinities between them. (**a**) Near-infrared iBright image of gel showed major band at ~ 65 kD which demonstrated NMI-protein binding interactions at NMI 1 µM. Lane 1 shows NMI and albumin incubated together for 24 h at 37 ºC, Lane 2 shows NMI, MAOA and albumin, all added at the same time and incubated for 24 h and lane 3 shows NMI and albumin pre-incubated for 30 min and then MAOA added to incubate for 24 h. (**b**) Black and white image of same gel. The amount of each protein added to the well is 1 µg. Gel was imaged using iBright at 750 nm (NIR wavelength) with exposure time 10 s for analysis.

To elucidate the selective binding affinity of NMI with both MAOA and albumin under low equimolar conditions (1 µM each), we tested three drug-protein binding conditions. To systematically investigate these binding relationships, NMI was incubated with albumin alone, resulting in a bright band (Fig. 5a, b), which confirms strong binding of NMI to albumin. When equimolar concentrations of MAOA, albumin, and NMI were mixed simultaneously, the NMI-labeled band was nearly undetectable in this condition, indicating that despite NMI’s strong binding to albumin, *in the presence of MAOA*, NMI preferentially interacts with MAOA. The reduced visibility of a band in Lane 2 was attributed to MAOA the result of equimolar albumin readily observed in lane 1 in the absence of MAOA (Fig. 5a, b) suggests these concentrations and amount loaded (1 µg of each protein) were below the threshold to visualize an NMI-labeled protein band for MAOA, although in the previous experiment, the MAOA band was detectable (Fig. 3). These equimolar conditions also ensured no excess NMI remained to interact with albumin. To further validate and understand these binding affinities, we pre-incubated NMI with albumin for 30 min before introducing MAOA and allowed the mixture to incubate for an additional 24 h. This approach revealed that NMI initially binds to albumin within the first 30 min; however, upon addition of MAOA in the absence of higher affinity MAOA binding protein, when the interaction is halted by MAOA (Fig. 5a, b). The resulting gel showed a fainter band compared to the 24-h NMI-albumin incubation, reflecting the initial 30 min binding period. These findings suggest that although NMI exhibits strong binding to albumin, it has a higher affinity for MAOA, as evidenced by the preferential interaction observed under competitive binding conditions. This preference of NMI for MAOA over albumin highlights its potential specificity and efficacy in targeting MAOA.

### MAOA Protein Identified by Mass Spectrometry within NMI ~ 65 kD Band from Colorectal Cancer Tumor

**Fig. 6:**
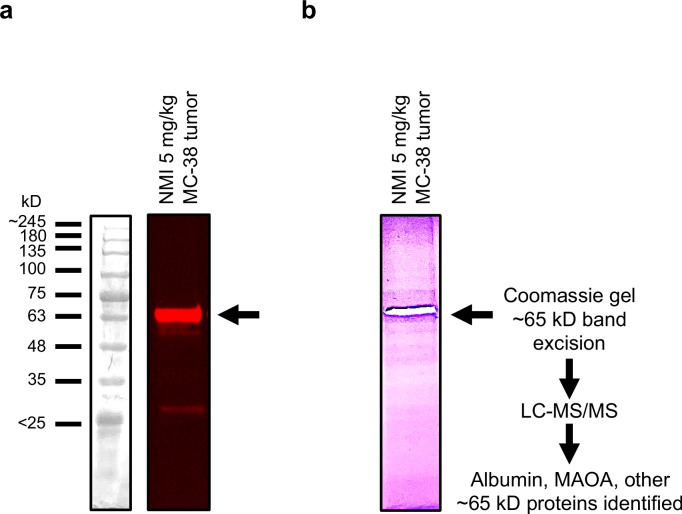
Tumor tissue lysates were prepared from mice bearing MC-38 subcutaneous colon cancer tumor treated with NMI (5 mg/kg, every other day; IP) for 21 d. Tumor tissue lysate (20 µg) was separated on SDS-PAGE gel. Gel was imaged using iBright at 750 nm (NIR wavelength) for exposure time 10 s for analysis. (**a**) Near-infrared iBright image of gel shows a major band at ~ 65 kD. (**b**) Coomassie stain gel image with band excised for LC/MS/MS protein identification.

NMI binding at ~ 65 kD was observed in MC-38-derived colon cancer tissue (Fig. 6a). The ~ 65 kD fluorescent band was cut from gel for mass spectrometry analysis to identify the proteins that may be associated with NMI (Fig. 6b). The most abundant blood protein, albumin, was readily detected in the ~ 65 kD NMI labeled band of NMI treated MC-38 tumor tissues with 54 unique peptide sequences identified from 658 peptide spectrum matches. Importantly, MAOA was also identified in the NMI-labeled band for MC-38 tumor samples. Two MAOA peptides were detected by LC–MS/MS in the 65 kD band of NMI treated MC-38 tumor tissues: [K].WVDVGGAYVGPTQNR.[I]; [K].INVLVLEAR.[D]].

## Discussion

NMI has been shown to reduce cancer growth of the prostate [14], brain [7], and colon [39]. This study demonstrated that specific proteins interact with NMI. MAOA KO mice compared to WT showed significantly less NMI imaging signal in whole animal and organs then further examined at the protein level in brain and liver which confirmed that MAOA is a major protein target. A ubiquitous background signal was observed in KO mice that was attributed to circulating NMI bound to its blood carrier protein albumin. The stark difference in signal intensity between KO and WT highlights the contribution of MAOA to NMI biodistribution which we previously examined extensively in WT mice [15]. We previously showed NMI biodistribution in many tissues of WT mice that correspond to MAOA expression, and indeed tissues with high MAOA such as lung, liver, kidney, and tumor had very high NMI signal whereas low MAOA expression tissues such as adipose and spleen had low signal [15]. In the current study, the same pattern of *ex vivo* organ fluorescence represented by brain and liver was observed in WT mice but relatively muted in KO.

In the immune-competent intracranial glioma model, we showed a distinct NMI-bound protein band around 65 kD in GL26 brain tumor lysates with high contrast to adjacent normal brain tissue after a 5 mg/kg dose. As a targeted theranostic molecule, the KO data predict NMI will be targeted to tumors with high MAOA expression, associated with high malignancy and chemoresistance, supporting its adjuvant utility. Based on NMI chemical structure and predicted molecular sizes of MAOA and its blood carrier protein albumin, the band contains both albumin and MAOA albeit at different amounts making it difficult to distinguish between albumin and MAOA expression. The more NMI added, the brighter the fluorescent label for albumin and thus the less abundant MAOA was visible but to a lesser extent in tissue samples. The complexity of biological tissue samples necessitated stoichiometric studies conducted *in vitro* with controlled pure protein samples.

To further investigate these NMI-binding interactions, we conducted a series of experiments to examine the two major binding proteins, MAOA and albumin, separately and in combination. It was found that when less protein sample was loaded to the gel (e.g., 1 µg/well), the signal intensity of the albumin band was optimal, however, the NMI-bound MAOA band loaded at the same molar amount was not readily detected. With 5 times more MAOA loaded (5 µg/well), the ~ 65 kD NMI-bound band became visibly apparent in the gel and corresponded with MAOA binding and enzyme inhibition, both greatly enhanced by PEG. Several possible molecular explanations are plausible including 1) MAOA is predicted to have only one high-affinity binding site for the NMI clorgyline triple bond moiety via reactive acetylenic inhibition known to form a stable interaction with N5 position of flavin adenine dinucleotide (FAD) cofactor of MAOA, binding to the enzyme active site stoichiometrically mol/mol of enzyme [40]. Albumin has several lower affinity general carrier binding sites and one cysteine predicted to bind covalently with the MHI-148 HMCD moiety of NMI, 2) albumin itself acts as a solubilizer or carrier, keeping the hydrophobic ring structure stable and fluorescent in aqueous buffer whereas MAOA binding to its clorgyline moiety may leave the NMI fluorophore exposed to the polar environment and susceptible to quenching [41], 3) NMI covalent binding to albumin could be more stable and readily detectable under SDS-PAGE conditions. Albumin has multiple binding sites including non-covalent Sudlow I and II natural hydrophobic binding pockets, 7 fatty acid binding sites, and a single free thiol on Cys34, a potential covalent site [42]. As the most abundant circulating plasma protein, albumin carries a wide array of drug molecules and nutrients to their high-affinity tissues and protein targets and thus has several potential NMI binding sites of varying affinities. Although, there could be intraspecies differences in drug binding in albumin protein from human, bovine, and mouse orthologs, the reactive Cys34 and its hydrophobic pocket thought to bind to MHI-148 moiety is relatively conserved [16, 21, 27, 43]. As a blood carrier protein, in addition to Cys34 there are potentially other albumin binding interactions to NMI that could be further defined in future experiments. In contrast, the intracellular enzyme MAOA has a well-defined single clorgyline binding site at N5 of the flavin cofactor within its catalytic channel with mol/mol stoichiometry [40, 44]. Under equimolar protein binding conditions (NMI:MAOA:albumin, 1 µM each), NMI was shown to have higher affinity for MAOA than to albumin as MAOA completely perturbed albumin fluorescence compared to NMI and albumin alone. When albumin was preincubated for 30 min with NMI followed by introduction of equimolar MAOA, binding to albumin essentially stopped but was not reversed, observed as a moderate fluorescent band compared to NMI and albumin alone. This preincubation experiment captured both NMI’s selective binding preference for MAOA and the extent of persistent binding to albumin formed within 30 min under *in vitro* controlled conditions with purified proteins.

When NMI was administered to a mouse model of colon cancer, both albumin and MAOA were detected by mass spectrometry within the fluorescent ~ 65 kD band of tumor lysate. Although MAOA is the preferred target, NMI must first pass through the blood via albumin, enter the cancer cell, and bind MAOA on the outer mitochondrial surface. Therefore, it is advantageous to have serum albumin first binding as a carrier to enhance drug-delivery then ultimately reach its target, the MAOA-expressing tumor.

Further confirmation of NMI affinity for MAOA was demonstrated by dose-dependent MAOA inhibition. NMI inhibition of MAOA was enhanced several-fold by improving aqueous solubility using PEG as the solvent vehicle in the *in vitro* enzyme activity assay [45]. Further investigations are warranted to explore the clinical implications of NMI-MAOA interactions.

## Conclusion

This study aimed to identify protein binding interactions with the NMI, a rational designed anticancer molecule. Specifically, NMI was shown to bind MAOA and the blood carrier protein, albumin, providing insights to image and treat cancer.

## Data Availability

All data supporting this study are included within the article.

## Abbreviations

C57: C57BL/6 J mouse strain
EPR: Enhanced permeability and retention effect
HMCD: Heptamethine cyanine dye
IC_50_: Half-maximal inhibitory concentration
KO: Knock-out *Maoa* gene mouse model
MAOA: Monoamine oxidase A
NIR: Near-infrared
NMI: Near-infrared monoamine oxidase inhibitor; MHI-148 clorgyline
PBS: Phosphate buffered saline
PEG: Polyethylene glycol
ROI: Region of interest
SDS-PAGE: Sodium dodecyl sulfate polyacrylamide gel electrophoresis
SPARC: Secreted protein acidic and rich in cysteine
